# The underrepresentation of women in sport leadership in South Africa

**DOI:** 10.3389/fspor.2023.1186485

**Published:** 2023-12-13

**Authors:** Nana Akua Achiaa Adom-Aboagye, Cora Burnett

**Affiliations:** Centre for Sport Leadership, Maties Sport, Stellenbosch University, Stellenbosch, South Africa

**Keywords:** feminism, gender equity, South Africa, sport leadership, global South

## Abstract

**Introduction:**

The lack of representation of women in sport leadership, despite global movements and policies that have found some traction, is a persistent, unremitting challenge globally, and especially in South Africa. This study aimed to explore the intersections of gender and sports ideology and its impact on gender (in) equity in the South African context. The study draws on African feminist theories and perspectives as a conceptual framework.

**Methods:**

Twenty-eight interviews with prominent administrators, gender activists in sport, and practitioners from the sport-for-development sector and thematic document analysis provided qualitative data for the generation of three main themes relating to: (i) norms and values; (ii) male resistance; and (iii) agency.

**Results:**

The results of the study show minimal traction on changing patriarchally informed cultural beliefs towards women with men as gatekeepers and masculinity framed for leadership attributes in most sports.

**Discussion:**

Within an African feminist viewpoint, gender justice is multilayered and the inclusion of women within a holistic environment of shared decision-making and equitable resource mobilisation and distribution cannot be achieved through advocacy alone but necessitate the mainstreaming of a gender agenda to meaningfully address transformative change of sport systems and practices.

## Introduction

A socio-political reality underpins the ecology of sport that reflects a sex-binary perspective based on constructed physiological and biological differences. The “sexes” remains entrenched in identity-defining patriarchal ideologies, and cultural perspectives that translate into various manifestations of sexism. In recent times feminist movements have experienced increased global mobilisation of their advocacy, whilst simultaneously challenging various forms of resistance within society across all stakeholder types. Such challenges are often entrenched in Western feminist approaches and praxis, which women find foreign to an African perspective that seeks reconciliation and integration in a non-confrontational approach. Perspectives that can be engulfed in racial and patriarchal struggles, despite the democratization of African governments.

In Africa, deeply-entrenched patriarchal systems continue to be resistant to international gender-related reforms and feminist actions, especially amongst more culturally traditional and rural populations. A woman's perceived role, stereotypical portrayals, and responsibilities as a caregiver have led to consistent, unequal, and unfair dispensation for women and girls in all spheres of their lives (1). Yet, womanhood is embodied in the African worldview as opposed to a western-informed perspective. Feminist praxis focusing on radical reform and justice of global influence posit gender as a more fluid and non-binary phenomenon, as scholars and activists challenge the status quo and advocate for significant reforms within sport and broader society (2).

Globally, sport remains a sector entrenched in male-dominated ideology, structures, and practices where women continue to work in the shadows of male gatekeepers, especially within decision-making roles, which continue to be influenced by embedded patriarchal structures. The relative absence of women in sport leadership has sparked notions of gender reforms in this domain. Organisations like the Association of Summer Olympic International Federations (ASOIF) investigate and expose unbalanced executive board membership by revealing a female membership range, which currently stands between 40% (three federations) and under 15% (five federations) (3). Existing gender inequalities find heightened expression in sport, particularly at decision-making levels, despite documents such as the *IOC Gender Equality Review Project, Olympic Agenda 2020: 20 + 20 Recommendations* and the *Olympic Charter* and similar initiatives making some inroads in addressing gender transformation in sport leadership (4–8).

Regionally, the African Union (AU) has recommended the promotion of women in sport leadership (9). This is further advanced by the AU's endorsement of the Kazan Action Plan (KAP)—a document endorsed by most national governments, committing sport policy development to the UNs Sustainable Development Goals (SDGs). The KAP calls for the enforcement, advancement, and empowerment of gender equality for women and girls in sport (10, 11). Such an endorsement commits the AU to implement policy, guide gender reform and emphasise action plans following declarations concerning proposed reform (10). This has further contributed to National Olympic Committees (NOCs) having gender commissions and implementing reporting mechanisms to address gendered leadership reform.

Despite the formulation and apparent implementation of gender reform policies and targeted leadership reports, men still predominantly hold power within sport and society and act as the main decision-makers (12, 13). In Africa, this hegemonic masculinity enshrouds women's access to resources and fight out of poverty, whilst seemingly nurturing continued male dominance (14). Within sport this has contributed to uncomfortable electoral processes for some women who stand for leadership positions. These women will face multiple obstacles and resistance from men who have been dominating executive boards and created an exclusionary organisational culture (boys club) (15, 16).

This is particularly true of traditionally male sports, where if women are awarded positions, it is usually that of secretary or chairing gender or safeguarding commissions. This contributes to apprehension from some women who are critical of tokenism for compliance's sake and who aspire to future leadership roles in sport (17). This type of hegemony has settled as an accepted status quo within and across the structures of many sport organisations (global to national), lending itself to the unspoken belief that successful (sport) leadership rests in ascribed masculine competencies, influenced by socio-political processes and practices (18). The underrepresentation of women increases as one moves from participation levels to leadership positions. Often, especially in Africa, there are no clear pathways to guide players to administrative and ultimately leadership roles (4, 19, 20). This alludes to the unequal power relations which continue to exist at the highest levels of sport, where proposed gender-equality targets at the executive level are still far from parity. The best example of this is at international level, where the International Olympic Committee (IOC) has proudly recommitted itself to ensuring 50% female representation at the participation level, but only 30% female representation at decision-making levels (21) for the upcoming Paris Games (2024). Such a statement by the IOC can only realistically be achieved if independent agencies such as NGOs and NOC stakeholders (government and private sector organisations for example) are allowed to provide the necessary support and guidance at ensuring gender parity.

The study is framed within an African feminist paradigm as an alternative to feminism, allowing for the unpacking of current gender practices (during interviews) to explore existing and emerging discourses and influences emanating from lived experiences within South Africa.

The focal point (aim) of the study was to explore the experiences and opinions of those involved in sport in South Africa (civic society and/or activists), regarding issues of fairness and justice for women and girls (22), whilst acknowledging and appreciating the various intersectionalities of each participant in the South African context. This paper reports on specific research findings of the above-mentioned study, that revealed insights that underpin the current gap in women in sport leadership, by providing intersectional reflections and experiences on ongoing hegemonic practices and structures experienced in South African sport.

The representation of civic society and activist or reformist perspectives feature agency as an important dimension. A dimension that is often amiss from Western perspectives and scholarly output that homogenise women in sport leadership challenges. Thus, a different paradigm informed by Southern Theory as propagated by Connell (23, 24) may meaningfully inform current global gender equity discourse, especially framed by activist perspectives from civic society (sport for development or grassroots sport development) and sport sector (25). A discourse that needs to recognise and mediate marginalised voices from developing southern nations (global South), as indigenous knowledge production interfacing with modern (and often exclusionary) knowledge practices and reproductions from the developed and wealthy nations (global North) (26).

## Women in sport leadership

Although studies have acknowledged the lack of women in sport leadership and that multiple barriers exist, there is a continued lack of in-depth understanding of the current situation (13, 27, 28) within developing contexts, and of the identification of contextually-informed challenges, barriers and opportunities (29). This could be attributed to the apparent disconnect between practice and gender equality/equity policy frameworks, resolutions, and strategies from entities such as the IOC, UN Women, AU, African Union Sports Council (AUSC) and declarations from entities like IWG Women and Sport that provide guiding principles for ensuring gender reform.

Various scholars have identified barriers (discrimination, harassment, stereotypes, access, and treatment) to and causes of the global underrepresentation of women in sport leadership positions, relating them to gender-biases in recruitment and/or election processes and gendered beliefs of required skillset(s) for success (18, 30, 31). Subtle, discriminatory practices have also been identified as impeding women's progression within an organisation (32).

Feminist scholars and women in sport-related social movements have often presented research and positionality that frame global feminist discourses as representative of all women's experiences and plight to strife, regarding access to sport leadership. Yet when one examines these assertions through the lens of geopolitics, one discovers that the work is country and region (global North) specific (33, 34). A more in-depth critical analysis is essential to provide root causes and insights beyond the positivist surveys of regional studies (4) by scholars from the north. Thus, academics and activists from marginalised positions who create rather than merely consume and augment existing paradigms, should be supported when attempting to address this gap in mainstream gender discourse.

### Women in sport leadership in Africa

Identified issues presented in “global” women in sport leadership studies are not always transferable, nor relatable to marginalised women from developing contexts such as Africa (35). There have been scant studies that have identified similar, (albeit at times differing) issues impinging on women in sport leadership from an African perspective from the formal sport sector. However, experiences of patriarchy differ between various societies and often within the same society, as race, culture, religion and socio-economic status intersect in a myriad of ways (36–40). Amongst these differences, race is the most prevalent of socio-political constructed labelling of people within society. It is this logic that influenced notions of colonial superiority and post-colonial thought on stratification of manifestations of race, gender and other constructs. However, Africa's (especially South Africa's) structures, systems and decision-making continue to be permeated by the biological characteristics (outward physical differences) reminiscent of colonial power structures (41). Thus, this study refers to the concept of “race” as a socio-political structured concept that influences real-life experiences of people with identifiable racial characteristics overlayed by gender, to position them within a hierarchical order of dominance or subjectivism in society and in sport.

In most traditionally African societies and/or communities across the region, a woman's place is influenced by ideological and socialisation practices in traditional societies in the region. This contributes to women being first and foremost viewed as being responsible for child rearing and homemaking (42). Even when a woman can attain a leadership role, it is often fraught with resistance and obstacles not only from the home but from the organisational culture of a sport organisation and its environment. A lack of role modelling, mentorship, and support (and even respect) has been identified as the norm for African women in leadership positions (43, 44). The (in)ability to connect and engage with prominent women in sport (45) can also be viewed as discouraging towards women who aspire to sport leadership roles in Africa (44).

Atanga (46) provides a convincing argument for such experiences, by stating that “traditional practices are also arguably perpetuated and sustained by ideological brainwashing, through hegemonic patriarchal practices and discourses”. The lack of appropriate and accessible education (which acknowledges and respects the heterogeneity of African culture and traditions), that promotes gender equality/equity according to a human justice framework, is considered a systemic barrier that exists in most social institutions (44).

Gender, intersecting with ethnicity/race (contextualised by country), religion, culture, and class, can add various layerings of social stratification linked to different levels of disadvantage associated with the entry of women into sport leadership positions. This includes not being able to access financial resources for professional studies and not having access to supportive networks (social capital), for executive leadership positions (38, 45).

Such layering of society, especially within the South African context of poverty and the post-apartheid dispensation (race relations), has special meaning for women wanting to enter sport leadership positions, as it can impede upon their access to resources and support, which ultimately influences who is able to enter leadership. This is why it is also important for the media to provide adequate and fair coverage of women in sport leadership. However, the media's profiling of women in sport leadership (and the challenges they face) can at times be highly biased, not only in South Africa (47, 48) but in Africa as well (43, 49).

## Conceptual framework underpinning the study

In South Africa, the leadership landscape for women in sport can only be described as “dire”. Research in this field is limited (4, 50–52), with only a few studies focusing on the overall gender equality/equity landscape within a sporting context as it mainly relates to participation (53–55). Comparing the handful of recent studies (56, 57) with earlier ones (58, 59), indicate that not much has changed since democracy (1994), when South Africa held its first democratic elections post-apartheid (institutionalised racial segregation by the government—1948 to 1994). A regional study in 2014 and its follow-up in 2021 highlight slow progress for women to access leadership within the formal sporting sector as guided by selected targets of the United Nation's (UN) policy framework and that of the AU (4, 50). The above-mentioned studies will help to frame the skewed sport leadership landscape in South Africa, within an African feminist context.

### Skewed sport leadership landscape

The gender leadership composition of South Africa's three most popular sports (by numbers) aligns with the assertion from the limited studies already conducted and confirms that sport leadership in South Africa is inherently male-dominated and in need of urgent reform (51, 52, 60). For example, at the time of writing, the current gendered leadership composition of the three leading sports in South Africa—cricket, football, and rugby—constitute male dominancy (61–63):

• Cricket has about 13% female executive board representation (2 out of 15 board of directors);
• football has about 19% (4 out of 21 executive committee representation) and
• rugby has about 36% female executive council board representation (5 out of 14 members).

It is evident that the South African sport leadership landscape is not being rigorously monitored by the Eminent Persons Group on Transformation in Sport—the assigned national task team overseeing racial and gender transformation in the country. The Eminent Persons Group's mandate is to annually audit and report on transformation in South African sport, in alignment with the prescripts of the *Transformation Charter for South African Sport*. The *Transformation Charter* is a guiding document for an equitable and demographically representative South African sporting landscape (64).

The most recently available *Eminent Persons Group Report: 2018/2019* focuses more on racial transformation, leaving reporting on gender transformation at the periphery. The report provides gendered statistics (continuously low) and does not provide actionable recommendations for stakeholders to combat this (65–67). Policy reform and decision-making within leadership require conceptualised guidelines and not target-setting accounts, as is the current norm in South Africa. Such guidelines need to consider the intersectionality that South Africa's diverse population experiences, especially as remnants of apartheid still exist within South African sport structures (68).

The skewed gender representation of women in sport leadership that contradicts the growing participation levels (13), is a matter of concern. The value of this study lies in the fact that it presents an indigenous perspective that seldom features in dominant literature, as evidenced in the systematic review conducted by scholars (69) stating the over-representation of studies from a liberal “Western” tradition and absence of the nuances of intersectionality (27, 28, 70). Indigenous voices, especially as it relates to race and gender in a post-colonial (and apartheid) setting grappling with deepening social inequalities and the contextual interpretations thereof, affect how sport ultimately operates in the country and its locales (18, 29, 53).

### African feminist lens

Critical African feminism has been used to underpin this study as appears in the work of Stiwanism and Nego-feminism (sub-branch of African feminism). Stiwanism is derived from the term “Social Transformation Including Women of Africa” (STIWA) and posits that men and women need to acknowledge, collaborate and address social inequalities, to meaningfully inform and contribute to social transformation. This requires the facilitation of a shift and change in mind-sets (69). Nego-feminism stands for “no-ego” feminism and argues that for gender reform to be established, men and women need to come together, set their egos aside and respectfully negotiate through their issues, especially pertaining to patriarchal structures so as to achieve equitable solutions (72). This speaks to the mainstreaming of gender inclusion (men and women) in decision-making and leadership roles. Critical African feminism is meaningful as it allows for the capturing and framing (from a civic society and activist perspective) of women aspiring to sport leadership in South Africa (22). African feminism is about discerning the unique lived experiences of African women within patriarchal systems during and after the major trends of colonialism (and apartheid in South Africa) (73). Understanding how these influences and experiences affect women (past and present) and intersect with oppressive traditions, cultures, structures, and practices (74), provides a holistic view of the sluggish attainment of gender equity, despite national alignments, “commitments” and ratifications of international gender policy (75, 76).

## Methodology

The study was of an exploratory nature and was influenced by African feminism, Stiwanism and Nego-feminism (as discussed above). These provided a yardstick with which to understand and measure the relayed experiences, behaviour, and action(s) that were shared by participants (77), to assess their merit in alignment with the research objectives.

### The research

The study followed a qualitative, descriptive design informed by critical insights from African feminism. Policies and national strategic plans were scrutinised as part of the thematic document analysis to capture all relevant gender-related information. This allowed for the verification of information obtained through interviews. It also informed interviews, which were the main method of data collection. Desktop research and initial interviewing of prominent male and female leadership in sport (stakeholder) organisations and sport for development (SfD) NGOs, contributed to the purposeful identification and selection of participants according to their knowledge, expertise, and experience with working with women and girls in sport (78). This was to ensure an unbiased reflection of women in various (sport and SfD) leadership roles. SfD NGO representatives were included in the data collection due to their agency as civic society representatives, where their advocacy and social movement(s) of advancing women in (sport) leadership, contribute towards a collective voice of resistance and power.

### Sample

The participants selected were representative of both genders, and various races (Black African, Indian, White, and Multi-racial) and worked in organisations that were spread across six out of the nine South African provinces. As previously mentioned, race, in the context of this study, shall be viewed as a socio-political construct built on biological traits due to the remnants of colonialism and apartheid within current social and sport structures. The term “multi-racial” stated above, also includes the racial classification of “Coloured”, in the South African context. For the purposes of clarification, the term “Coloured” used in this article is a racial classification in South Africa for individuals of mixed ethnic EuroAfrican descent.

There were 38 potential participants (civic society and/or activists) initially identified, and contacted via email (28), phone calls (4), Twitter (1), WhatsApp (1) and spoken to in person (4). Of the 38 initially identified, 28 (18 women and 10 men) agreed to be involved in the study, six gave tentative confirmation to take part but were later unreachable, two declined immediately and two did not respond to communication attempts at all. Of the 28 that finally partook in the study, 21 were from civic society (sport stakeholder representatives and SfD NGOs) and 7 were activists (sport administrators involved in the management of sport and promotion of women and sport). As previously stated, all participants had knowledge, expertise, and experience with working with women and girls in sport. All forms of communication were conducted in English as this is the first language spoken by the researcher. Despite South Africa having 12 officially recognised national languages, all participants were proficient in English and had no issues with the interview process.

### Research process and procedures

Semi-structured interviews were utilised as the main medium of data collection to form the foundation for dialogue and a shared connection and some trust [between the researcher and participant(s)], which encouraged the participants to freely engage and candidly express themselves regarding their experiences, issues, and ideas (78) that they felt were relevant to the study. Of the final 28 research participants, nine engaged in face-to-face interviews at their offices or public venues of their choosing, ten were interviewed via Skype (video call), six via WhatsApp call, two provided written responses via e-mail and one as per telephonic interview. All interviews lasted between 60 and 90 min and permission was granted by the interviewee for audio recordings.

### Data analysis

Creswell's (78) six-step data analysis process was utilised for theme generation. After transcribing the interviews verbatim, the data was imported into Atlas.ti for analysis and coding, using thematic analysis to identify common and emergent themes. This was done by reading through the data and assessing the general views, opinions and ideas. The next phase of coding entailed the creation of main and sub-categories. This initially generated three main and three sub-categories by utilising semantic demarcated content under appropriate labels (79).

### Trustworthiness

Trustworthiness includes the criteria of data collected to be credible, transferable, confirmable and dependable (80, 81). The credibility of the data was obtained through the representativeness of the research participants in terms of gender and roles within different organisations (81). Transferability necessitated detailed descriptions of the research process, whilst confirmability required objectivity from the researcher, with respect to her insider perspective. To ensure her personal socialised experiences would not incur possible biases, she shared results and themes with her research assistant and eventual doctoral supervisor. An additional concerted effort was made to present the experiences, opinions and views of participants during the analysis of the data for themes, by presenting information as it was recorded, so that participants' responses were accurately captured (80).

For the co-creation of knowledge, the researcher engaged in further discussions with research participants to counter possible biases, check meanings and ensure the accuracy of interpretation related to context (81, 82). Methodological and sample triangulation contributed to increased trustworthiness (82, 83).

### Ethics

The study received ethical clearance from the Faculty of Health Sciences (HDC-01-107-2017) of a public South African university. A high level of ethical conduct ensured that research participants remained anonymous without any breach of confidentiality. Pseudonyms were used throughout the interview process, ensuring that no references to the identities, designations, or roles of participants were made.

## Results

Research participants shared various examples during interviews, which highlighted patriarchy as a common thread within sport, linking identified themes, and an ongoing societal issue in South Africa (84). This is indicative of inter-generational, male-biased, and hegemonic ideology (influenced by colonialism and apartheid), which has become enculturated and entrenched through socialisation practices and gendered-role constructions—with sport often reflecting societal issues and challenges within the country. Such views (or opinions) and examples of micro-aggressions may be indicative of the fact that despite what has been enshrined in South African policy (gender equality/equity), some South African men (especially in sport leadership) continue to fear the assumed loss of their established (post-apartheid) power and privilege that gender equity may bring (85). These produced three emergent themes in relation to women in sport leadership, namely: norms and values for behaviour; male resistance and agency: redress. These three themes shall be presented below, to denote the socio-cultural and ideological underpinning and intersectionality (race and gender) that create barriers to women in sport leadership in South Africa.

### Norms and values for behaviour

The gendered expectations and patriarchal-inspired norms that continue to permeate South African society and especially Black African communities are not unique to marginalised communities. The research data provided evidence that despite national policies on equal opportunity for women in sport, gendered mindsets and expectations have superseded formal policy. The study determined that the ideology discussed above was pervasive in organised sport settings. Seasoned sport administrators/activists introduced the issues here. Their views provided an understanding of how gendered ideology in South African society seems to continually control women in sport leadership (behind the scenes), despite the public veneer of cooperation. Thus, indicating an underlying layer of fractured negotiations and collaborations between men and women within sport leadership.

Participant One (1) who was involved in sport for more than 40 years, explained how gender-related issues played out in the boardroom. She mentioned how she was often the only woman in the room during executive meetings, where men in charge usually relegated issues about women in sport to the final issue on the agenda. She explains:

I go to the meetings, it's all men and they want to talk about this and that and when it's my turn, half of them go to the toilet. The others fall asleep; it's useless! I fight when I go there but am a lone voice there. I just don't know if it's ever going to change, I am not confident.

Participant 1′s explanation alludes to the concept of women oftentimes being viewed as tokenized in South African society. Where women are placed in leadership positions, to “showcase” gender representation, rather than being actively engaged with on issues concerning gender reform. Her experience insinuates the lack of respect and acceptance from some men at the board level—an attitude that is widespread in competitive sport. She also believed that it is an ongoing struggle that is never-ending.

Participant Two (2) had similar sentiments and stated that, “Where there are women appointed somewhere in sport, you will find that they are not treated with respect [by men]”. She experienced resentment and criticism for being “too emotional or sensitive”. Yet it has not deterred her and often, their (men) biased opinions have justified her efforts to voice her opinion and take up leadership roles in sport organisations.

### Male resistance

For many of the research participants, the issue of how South African society views Black African and Coloured women and their place within their respective communities was of concern. Patriarchal gender ideology of a woman's place is often defended as necessary in maintaining Black African cultural practices—practices that were egalitarian before colonialism (86, 87). Women within more affluent communities (mostly inhabited by the White population from European decent), were seen to have more freedom and opportunity than their racial counterparts. Black African and, to some extent, Coloured (racial classification in South Africa, explained above) women, were pinpointed as struggling the most in terms of issues of gender equity and personal autonomy, in South African sport.

Several White research participants admitted that it frustrated and even frequently angered them to have to observe such discriminatory views, sexist portrayals, and exclusionary practices in the sport environment. Such observations were confirmed by Black African women who confirmed that their “culture” “expected very little from women” (outside the home), especially in the public domains regarding leadership roles or even basic employment capabilities.

Participant 2, who has been involved in sport for over 30 years, and served on the boards of multiple sports, believed that such a view is informed by ideology:

“It is mental and that is why I am saying it's tools that we need. To say how do we then start this work and come up with tools [to change] the mindsets of men and women?”

According to her, world views in many communities, especially marginalised communities, have not changed despite a liberal constitution (South Africa) where human rights are prioritised. She stated that amongst Black African households, men control women—behaviour that transfers to boardrooms, where Black African men dominate decision-making in sport. The above is indicative of a need for shifts and changes in the mindsets of (Black African) men in South African sport leadership, as egos are seemingly driving decision-making involving women in sport leadership.

### Agency: redress

Most research participants proposed collective agency and that men should engage in gender-related issues. Research participant Four (4) (male) said:

 “There is a need to come together, to put issues aside and to be on an equal base. This is in order to provide equal opportunities for women as well as male participants”.

He believed that male leaders should implement gender policies, as all leaders should “acknowledge and realise the need for gender equity” (providing equal treatment, opportunities, and access based on need), instead of equality. Gender inequality is a societal and managerial issue where men should not be viewed as “the enemy”, but that merit should count rather than tokenism, for the sake of “ticking a box”. The question of capacity-building for women to take up leadership in male-dominated sports also remains contested.

The topic of women engaging with men to achieve gender equity, especially at board and administrative levels, led to some pointed responses from female research participants. Participant Five (5) and Participant Three (3) had the following to say:

“We have to educate men to give women the space, to be able to allow women to take their positions, as supported by law”. (Participant 5)

“There's still a long way to go, but there is education that still needs to be shared with our men about the differences and similarities between the two genders and the power I think that women have in the field, and also the lessons and learning that they can share. So that we can educate each other and be better”. (Participant 3)

Participant 5 believed that the reason that men do not adhere to policies in South Africa, is because they are not monitored, nor are punitive actions taken for transgressions outside the prioritising of racial transformation. Black African men especially, generally do not view women as equals from a cultural and societal perspective and despite legislation, they do not have examples or a prescribed code of conduct to follow.

Participant 3 believed that driving diversity should be a standard practice of good governance, with the collective (men and women) pulling of knowledge and resource sharing, to build resistance and reflection. This as societal influences, public opinions and cultural beliefs continue to inform the continuation of gender inequalities at all levels of sport that mitigate against women's access and promotion into decision-making positions (88).

## Discussion

Male hegemony finds global expression in behaviour by men in broader society and in sport (34). Yet regionally, African feminism, Stiwanism and Nego-feminism provide an additional layer, by acknowledging that Black African men may actually be reluctant to share their power and privilege with women, due to the acquisition of this by many, post-colonialism and post-apartheid (69–73). Such behaviour could therefore be linked to a lack of objectivity, based on research participants' shared experiences of uncoordinated and often unsupportive environments and negotiations found at the leadership level within South African sport (72).

Sport federations and organisations have made public commitments to gender equity in sport in South Africa (89), yet the experiences presented have indicated a different reality at the decision-making levels. As this paper has tried to show, the continued gender inequity issues in South African sport leadership, may be due to ingrained patriarchal ideology, with spin-offs resulting in male-dominated structures and male-biased practices that have their root causes in the colonial and apartheid subjugation of men of colour, Black African men in particular.

The findings presented have shown that gender ideology is unfortunately deeply rooted in various cultural norms and practices in South Africa, which have not kept up with changes to and adaptations of the interpretation of gender identity, norms, practices, and expectations that have taken place elsewhere in the world (44). The exclusion of women from leadership roles, has been deemed necessary in some quarters by men, to retain cultural beliefs and expectations, especially within the Black African community (90).

Despite South African sport legislation supporting the pursuit of gender equality/equity in sport (64, 91–93), women in sport leadership positions remain a minority. Many women still experience marginalisation and unequal treatment in South African sport (84, 94). Some research participants shared how they believed that it is often also how the media portray and prioritise male role models and male-dominated sports like rugby, soccer, and cricket. This indoctrination may have a bearing on the lack of female role models within sport leadership (95, 96). Men are still seen (and still consider themselves) to be the custodians of influence, power, privilege, and resources in the country when it comes to sport leadership (97).

Some of the current ideology is still entrenched in post-colonial/apartheid notions of neoliberalism, placing the onus of the liberation of women solely as women's responsibility, with disregard for widespread gender discriminatory structures and practices not under their control (74). Cognisant of South Africa's unique (racial) context and various cultures with entrenched gender beliefs, gender needs to feature prominently on the transformation agenda for societal change, and not merely be lobbied for by female activists or be part of political rhetoric around election time or during some other (limited) occasion(s) (72, 98). It takes political will and cognitive acceptance of Africa's egalitarian past, to meaningfully act on transformative legislation and bring real change beyond the current dominance of racial reforms in a post-apartheid era of redress.

## Conclusion

Patriarchal ideology and manifested hegemonic practices dominate most sport structures in South Africa (46) and contribute to an unfair gender environment, despite human-rights policies and political rhetoric. This hegemony has a consistent influence, especially at leadership levels within the sport sector. The lack of women in key decision-making positions and structures has had a snowball effect on how resources are allocated to women and girls in sport. However, it should not be about the gender of the decision-maker, but about their gender-mindedness, as men have a significant influence when it comes to ensuring gender equitable resource allocation and shared leadership. The increased inclusion of women in sport leadership positions is required if actions are to be implemented that truly achieve intended outcomes of gendered policies—including clear guidelines and appropriate rationalisations (99).

The study reflected on female experiences and insights from men and women with varied experience within and across various sport structures and entities, that highlighted the disconnect between policies and their implementation, as they relate to issues of gender and women in sport leadership in South Africa. The identified disconnect between policy and the implementation thereof (from policy to practice), was attributed to societal issues which are influenced by institutionalised male hegemony and African patriarchal thinking (100, 101)—both somewhat ensconced by colonialism and apartheid. This has resulted in continued experiences and practices of gender inequities at sport leadership levels.

Culture is used as the rhetoric to substantiate the current success (and failures) within leadership in South African sport. The celebration of a handful of women attaining top leadership positions (102) in South African sport is seldom more than allowing their voices in the public space, whilst simultaneously marginalising and oppressing others (103, 104). This study has also shown that the debate surrounding women in sport leadership often fails to detail barriers (the why) affecting gender inequity, especially in a country like South Africa. Thus, further investigative research needs to be conducted in South Africa, Africa and across various other geographical regions in the global South, focusing on:

• Gender equity and women in sport leadership within various sports organisations (i.e., federations, government, NGOs, corporates)—a comparative analysis.
• Lessons of experience that women in sport leadership can learn from women in other sectors.
• Talent recruitment and nurturing for women to take their rightful places in all structures and levels of sport leadership in South Africa.

This will allow for the identification of core issues impinging on gender equity in different sectors and across various levels of women in sport leadership. South African women lack the support from global feminist movements and local praxis, whilst women's issues remain the concern of women as a local manifestation of accepting the status quo as inevitable. The literature within this study has highlighted the lack of concerted and coordinated efforts by African (sport) leaders to set an agenda for meaningful and sustainable gender equity and mainstreaming in a dispensation where profiling men and leaders largely remains uncontested (50). In competitive sport, men emerge as natural leaders with the acceptance of some women in the echelons of power. In sport for development organisations, men still occupy most leadership positions although the ethic of care ascribed to the value of female implementers and role models provide the bragging rights for the sector. Systemic change is not only a slow process, but hardly possible unless incentivised or enforced by international or national policy actors with the political clout to drive a gender equity agenda.

## Data Availability

The original contributions presented in the study are included in the article/Supplementary Material, further inquiries can be directed to the corresponding author.
